# The Anti-VEGF(R) Drug Discovery Legacy: Improving Attrition Rates by Breaking the Vicious Cycle of Angiogenesis in Cancer

**DOI:** 10.3390/cancers13143433

**Published:** 2021-07-08

**Authors:** Domenico Ribatti, Antonio Giovanni Solimando, Francesco Pezzella

**Affiliations:** 1 Department of Basic Medical Sciences, Neurosciences and Sensory Organs, University of Bari Medical School, 70124 Bari, Italy; 2 Guido Baccelli Unit of Internal Medicine, Department of Biomedical Sciences and Human Oncology, School of Medicine, Aldo Moro University of Bari, 70124 Bari, Italy; antonio.solimando@uniba.it; 3 IRCCS Istituto Tumori “Giovanni Paolo II” of Bari, 70124 Bari, Italy; 4 Nuffield Division of Laboratory Science, Radcliffe Department of Medicine, University of Oxford, John Radcliffe Hospital, Oxford OX39DU, UK; francesco.pezzella@ndcls.ox.ac.uk

**Keywords:** angiogenesis, anti-angiogenesis, bevacizumab, drug resistance, VEGF

## Abstract

**Simple Summary:**

Several anti-angiogenic drugs have been approved for cancer treatment, alone or in combination with other anti-tumoral agents. Angiogenesis inhibitors cause drug resistance, metastasis formation, and reduced delivery of chemotherapeutic agents, as a consequence of decrease of tumor vasculature. The endothelial cells as gatekeepers inspired a revisited interpretation of the vascular function in several malignancies.

**Abstract:**

Resistance to anti-vascular endothelial growth factor (VEGF) molecules causes lack of response and disease recurrence. Acquired resistance develops as a result of genetic/epigenetic changes conferring to the cancer cells a drug resistant phenotype. In addition to tumor cells, tumor endothelial cells also undergo epigenetic modifications involved in resistance to anti-angiogenic therapies. The association of multiple anti-angiogenic molecules or a combination of anti-angiogenic drugs with other treatment regimens have been indicated as alternative therapeutic strategies to overcome resistance to anti-angiogenic therapies. Alternative mechanisms of tumor vasculature, including intussusceptive microvascular growth (IMG), vasculogenic mimicry, and vascular co-option, are involved in resistance to anti-angiogenic therapies. The crosstalk between angiogenesis and immune cells explains the efficacy of combining anti-angiogenic drugs with immune check-point inhibitors. Collectively, in order to increase clinical benefits and overcome resistance to anti-angiogenesis therapies, pan-omics profiling is key.

## 1. Introduction

Several anti-angiogenic drugs have been approved for cancer treatment, alone or in combination with other anti-tumoral agents, and anti-angiogenic therapy is essentially an anti-vascular endothelial growth factor (VEGF) or anti-VEGF-receptor (VEGFR) therapy [1]. The first anti-angiogenic drug, bevacizumab (Avastin), a humanized anti-VEGF-A monoclonal antibody, was approved for the treatment of previously untreated metastatic colorectal cancers in combination with chemotherapy [2]. Ranibizumab is a humanized antibody based on a single antigen-binding site (Fab) derived from bevacizumab, but with a higher VEGF-A binding activity. Tyrosine kinase inhibitors are additional anti-angiogenic drugs, which interfere with VEGFR-1, VEGFR-2, platelet derived growth factor receptor (PDGFR), fibroblast growth factor receptors (FGFRs), and Tie2 signaling [3]. VEGF-trap protein aflibercept, obtained by fusion of VEGF binding domain of VEGFR-1 and R-2, which acts as a ‘VEGF ligand trap’, has been approved for the treatment of metastatic colorectal cancer [4].

Anti-angiogenic drugs lead to an increase in patient’s overall survival (OS) in the range of weeks to months and a 3–6-month increase in progression-free survival (PFS), followed by relapse in tumor angiogenesis and growth. Discontinuation of the therapy is the principal factor responsible for the ineffectiveness of the anti-angiogenic therapies [5]. In fact, when VEGF-targeted therapies are discontinued, tumor vasculature is rapidly re-established [6], whereas continuation of bevacizumab treatment is associated with an increase in OS [7].

Angiogenesis inhibitors are responsible for metastasis formation and reduced delivery of chemotherapeutic agents, as a consequence of decrease of tumor vasculature. Increased invasiveness is secondary to enhanced expression of angiogenic cytokines or recruitment of endothelial progenitor cells (EPCs), favoring the formation of a pre-metastatic niche [8,9,10]. Clear cell renal carcinoma (CCRC) cells and glioblastoma multiforme cells show a high metastatic potential after treatment with bevacizumab and VEGF inhibition [11,12,13].

Resistance to anti-VEGF molecules is responsible for the lack of response and disease recurrence. Acquired resistance develops as a result of genetic/epigenetic changes conferring a drug resistant phenotype to the cancer cells [14,15,16]. The intent of this literature review is to uncover the state-of-the-art understanding of the key mechanisms supporting angiogenesis and facilitating an immune-tolerogenic environment throughout tumor growth and progression.

## 2. Vascular Normalization and Tumor Hypoxia

VEGF inhibition normalizes tumor vasculature, decreasing vascular permeability and enhancing delivery of oxygen and drugs to intratumoral sites [17]. Vascular homeostasis is transiently restored during the first days of therapy. The improvement in tumor oxygenation takes place in the last 2–4 days after anti-VEGF treatment [17]. Hypoxia re-increases, inducing systemic secretion of other angiogenic cytokines, selects more malignant cells, able to grow in hypoxic conditions, and stimulates β1 integrin expression, a marker of resistance to cancer treatment [18]. Hypoxia-inducible factor (HIF) plays a critical role in resistance to anti-angiogenic therapies and is a survival factor used by cancer cells in a condition of oxygen deprivation. Moreover, HIF triggers epithelial mesenchymal transition (EMT) and metastasis [19]. Invasiveness is increased as a consequence of the production of pro-migratory proteins, including stromal cells derived factor-1 alpha (SDF-1α), hepatocyte growth factor (HGF), and pro-invasive extracellular matrix proteins [20,21]. Circulating EPCs move to hypoxic sites and contribute to the generation of new blood vessels, and the inhibition of VEGF prevents the mobilization of EPCs to the tumor site [22,23]. Hypoxia triggers the differentiation of tumor-infiltrating myeloid cells to M2-pro-angiogenic tumor-associated macrophages (TAMs), the recruitment of EPCs, genetic instability in tumor endothelial cells, and the selection of more invasive metastatic tumor cell clones, resistant to anti-angiogenic agents [24,25,26].

## 3. Role of Inflammatory Cells, Endothelial Cells, and Tumor Cells

TAMs and tumor associated fibroblasts (TAFs) are both involved in resistance to anti-VEGF agents. Blocking TAM recruitment is the winning strategy to overcome resistance to anti-angiogenic therapy. Both TAMs and TAFs promote tumor growth and angiogenesis through the release of growth factors and proteases. Tumors refractory to anti-VEGF therapy display an increased number of myeloid-derived suppressor cells (MDSCs) [27]. In addition to tumor cells, tumor endothelial cells also undergo epigenetic modifications involved in resistance to anti-angiogenic therapies [28]. Furthermore, the effect that the anti-VEGF treatment has on the cancer cell itself has been largely ignored and only recently has been receiving the attention it deserves. Studies like the one from Luo and co-workers [29] demonstrate a direct effect of VEGF on the actual cancer cell rather than the vasculature, although this is something not taken into account so far in clinical trials. The importance of VEGF is further supported by the findings that, in some cases, tumors cells can become more aggressive after treatment with bevacizumab. This is, for example, the case with human glioma cells in which bevacizumab treatment induces invasion through the activation of the beta catenin pathway [30]. Moreover, bevacizumab-treated glioblastoma patients have an increased relapse in comparison to bevacizumab-untreated patients, linked to an upregulation of c-MET and phospho-c-MET [31].

## 4. Alternative Mechanisms of Tumor Vasculature

Alternative mechanisms of tumor vasculature, including intussusceptive microvascular growth (IMG), vasculogenic mimicry, and vascular co-option, are involved in resistance to anti-angiogenic therapies [32,33], but again, anti-angiogenic treatments have been so far provided without evaluating whether the tumors are angiogenic or not or in which proportions a tumor is angiogenic or not angiogenic [34]. IMG, in which the capillary network increases its complexity by insertion of a multitude of transcapillary pillars, has been observed in different human tumors, including melanoma, colon and breast carcinoma, lymphoma, and glioblastoma [32]. The vasculogenic mimicry, in which tumor cells differentiate into endothelial-like cells, has been recognized in melanoma, glioblastoma, renal cell carcinoma, and breast, ovarian, and lung cancer [35]. Bevacizumab elicits vasculogenic mimicry leading to tumor escape and metastasis [36]. Vascular co-option, in which tumor cells co-opt and grow as cuffs around adjacent vessels, has been observed in liver metastasis of breast and colorectal cancer [36]. Bevacizumab-treated metastatic colorectal patients showed a limited response due to vascular co-option [36]. 

## 5. Pleiotropic Role of VEGF in Angiogenesis, Inflammation, and Immunity

**Figure 1:**
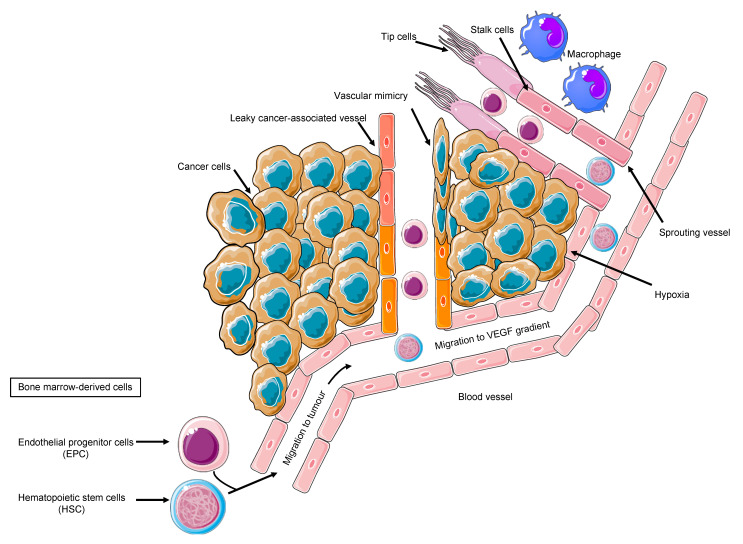
Cancer angiogenesis as a multi-faced, multistep process, recruiting divergent cell types and cell proliferation, migration, invasion, and differentiation, depending on the cancer-associated milieu.

Tumor cells, the secreted soluble factors the interstitial stroma and extracellular matrix, pericytes, and endothelial cells represent a complex neighborhood in which a vicious cycle develops, acting as a pro-angiogenic reservoir. The endothelial cells are main actors on the angiogenic field, expressing a plethora of tyrosine kinase receptors that trigger proliferation, migration, and differentiation signals [37]. Costa et al. uncovered Int-2 oncogene as an angiogenesis inductor [38] and prompted further studies on additional mechanism, including VEGF [39]. VEGF family comprises VEGF-A, VEGF-B, VEGF-C, VEGF-D, VEGF-E, and VEGF-F. VEGFR-2, also known as placental growth factor (PGF), is the main signal transducer and activates the main mechanism of PI3K, MAPK, IP3, and eNOS, which imprint one of the main features of malignant angiogenesis: the vasodilatation with endothelial detachment paralleling the cellular dynamics alteration [40]. The eNOS activation and NO production explain one of the major adverse events of anti-angiogenic treatment, namely the arterial hypertension, due to the decreased NO production. Conversely, VEGF production is related to vasodilation; VEGFR-2 is also related to cell survival and migration, and VEGFR-1 is additionally related to vascular stabilization, whereas VEGFR-3 is much more related to lymphangiogenesis [40]. By looking into the cellular system and compartmentalization for cell motility, it is important to highlight that an anaerobic metabolism is developed, and the proliferating endothelial cells (tip cell) trigger the sprouting angiogenesis via motile filopodia anchored to an extracellular matrix, attracting the cells through a VEGF gradient. Bystander cells suffice for an integrated system (stalk cell) while the cancer cell enhances the glycolytic activity supporting its migratory activity [41]. Thus, the actors on the scene are the neoplastic cells, the tip cells, rapidly migrating, the stalk cells, with supporting function, and the quiescent phalanx cells from which this structure is sprouting [41], tip and stalk cells being the most responsive to VEGF and its receptor VEGFR-2. The tumor vascular pattern largely differs from the normal vascular one in terms of morphology due to an abnormal vascular pattern of growth in which the blood and nutrient flow is absolutely aberrant, driving ischemia and abnormal solute and drugs delivery as demonstrated by VEGF expression tumor imaging performed with [42] Zr-Bevacizumab, with a decay in the drug concentration in 168 h. Contrariwise, as a proof of concept, VEGF within the tumor site is high. Imaging of VEGFR-2 expression addressed with [43] CU-DOTA-VEGF121 paralleled VEGF expression behavior in vivo models [44]. Remarkably, the role of hypoxia and VEGF in cancer is multifaced; nonetheless, in a hypoxic tumor, an overproduction of VEGF exerts an inhibitory effect on the dendritic cell and a stimulatory effect on the tumor-associated macrophages and on MDSCs, boosting Treg lymphocytes. Therefore, the result is an aberrant vasculogenesis within an immune-permissive niche [45] (Figure 1).

The expression of angiogenic factors in gastrointestinal cell lines behaves as an archetype of the spectrum of a new vessel-sustaining phenotype. Across the plethora of pro-angiogenic molecules, VEGF largely predominates over PIGF, interleukin-8 (IL-8), and fibroblast growth factor-2 (FGF-2) [46]. Remarkably, one of the paramount features of the tumor angiogenesis is the presence of precursors progenitors within the newly formed vessels expressing VEGFR-2. EPCs promote angiogenesis in hepatocellular carcinoma [47]. Ligand neutralization, antibody targeting extracellular domain of VEGFR, and small molecules’ tyrosine kinase inhibitors are valuable strategies [48]. However, the parameters involved in the dynamics of blood flow are crucial in order to modulate and normalize the pathological vicious cycle of angiogenesis. Amid the cancerous vascularization, shunts are predominant over perfusion, due to a high interstitial pressure halting the soluble factors diffusion within the tumor. Therefore, allowing a normalized perfusion and restoring the angiogenic homeostasis are optimal activity markers [49]. Moreover, a markedly elevated interstitial fluid pressure in human tumors and normal tissues decreases the drug effect [50]. Among the tumor diseases, the stronger rationale behind the use of an antiangiogenic therapy in case of kidney tumors is due to VHL gene mutation [11,51]; nonetheless, in gastric cancer, HIF-1 overproduction boosts pro-angiogenic factor production [52]. Indeed, gastric cancer represents an ideal model to discuss anti-angiogenic approaches’ challenges in oncology. VEGF and VEGFR are significant molecular targets in gastric cancer [53,54,55]. Furthermore, VEGF and PDGF production in gastric cancer is expressed in the different subtypes [56], holding biological implications since the Kaplan–Meier OS curve significantly differs in relation to preoperative serum VEGF levels [57]. Wang et al. corroborated these findings by stratifying patients according to type A vs. type C VEGF [58]. A synergic action between chemotherapy and angiogenesis inhibition is plausible due to the experimental model’s results showing that bevacizumab improves the penetration of paclitaxel and the antitumor effect on a MX-1 breast cancer xenograft [59]. Moreover, VEGF and HIF-1alpha are dose-dependently decreased by SN-38 in experimental models [60]. These results prompted an intensive investigation in order to better sketch the proper patient for the proper treatment. However, the adaptive–evasive responses by tumors to anti-angiogenic therapies represent an unmet need [48], due to the lack of validated soluble biomarkers, despite the intensive investigation also in the field of liquid biopsy [61,62]. A paradigm shift has been opened by the discovery of the glycosylation-dependent galectin-VEGFR-2 binding, which preserves angiogenesis in anti-VEGF refractory tumors [63]. This study initiated an intense investigation regarding efforts aiming to overcome resistance to angiogenesis inhibitors.

## 6. The Biology of Angiogenesis and Its Implication in Overcoming the Challenge of Identifying Biomarkers

Anti-angiogenic therapies have limited efficacy, only prolonging OS in metastatic colorectal carcinoma. Several different indications have demonstrated that primarily advantages in PFS have been obtained, and only in a small number of cancer indications including colorectal (mCRC), an increased OS has been observed (Table 1).

Indeed, some tumors are initially resistant to VEGF inhibition, and others eventually develop resistance. Two types of phenotypic resistance are classically observed. Intrinsic non-responsiveness includes cases of malignancies that at first do not respond to anti-angiogenics, yet a later adaptive response to treatment can be observed; hence, patients who initially obtain a response can relapse afterwards, showing adaptive mechanisms of resistance [64]. Within the intrinsic resistance to angiogenesis inhibitors, there is a pre-existing multiplicity of redundant pro-angiogenic signals. As soon as the tumor cannot be fueled by VEGF, an ability to circumvent this dependence is operative, feeding into a vicious cycle boosted by PGF, angiopoietins, and PDGF [65], activating several biological pathways such as VEGFR-2, mTOR, and heparin-binding EGF-like growth factor (HB-EGF)-epidermal growth factor receptor (EGFR) signaling [43,66,67,68] and acting as an intelligent evolving organism. The inflammatory cell-mediated vascular protection prompts the hypothesis and the notion of combining anti-angiogenesis with immunotherapies [11,22], because the tumor becomes hypoxic and growth inflammatory and immune-derived mediators unleash monocytes, macrophages, and MDSCs. All the above said cell types are able to produce angiogenic factors in both solid and hematological malignancies [69,70,71]. Thus, halting angiogenesis with anti-angiogenic compounds [72,73] and approved drugs [74], immunotherapies can parallel the “on-tumor” effect by hampering the anti-proliferative effects [75]. Additionally, a characteristic hypovascularity and indifference toward angiogenesis inhibitors has also been observed. The archetypic example is represented by pancreatic ductal adenocarcinoma (PDAC), where it is possible to detect a broad range of evidence of a poorly vascularized and fibrogenic area [76,77] with a characteristic cancer propensity to survive within a hypoxic milieu [78,79]. Consequently, PDAC is difficult to treat with anti-angiogenics; therefore, novel approaches have attempted to combine immune-directed therapies to peculiar invasive phenotypes [64,78,80]. Conversely, acquired resistance to angiogenesis inhibitors is mainly due to activation and up-regulation of alternative signaling pathways within the tumor, also supported by the recruitment of bone marrow-derived pro-angiogenic cells, such as the circulating EPCs and immune cells, which all have the ability to generate pro-angiogenic factors [81,82]. Moreover, increased pericyte coverage of tumor vessels is also important, particularly due to active PDGFR-beta signaling [22,83,84] also being an issue when we think about cardiovascular toxicity [85]. Surprisingly, the activation and enhancement of invasion and metastasis to provide access to normal tissue vasculature has also been observed in case of anti-angiogenesis therapy [86,87,88]. Nevertheless, this preclinical evidence has not been confirmed in a robust clinical fashion, with the exception of a report on glioblastoma [89]. Van Beijnum et al. reviewed the hallmarks of resistance to angiostatic therapy, systematically pinpointing growth factor redundancy, recruitment of bone-marrow derived cells, and local stromal cells as the main factors paralleling vessel co-option and vasculogenesis mimicry in jeopardizing anti-angiogenesis [14]. Moreover, endothelial cell heterogeneity can prompt MDR molecule expression [42], and extracellular vesicles have been shown to decrease the anti-angiogenic effects [90]. Specifically referring to sunitinib, lysosomal degradation [91] emerged as a novel mechanism of resistance, warranting an unexplored dual inhibition [92]. Additionally, glycosylation [63] and genetic polymorphism [93] represent emerging mechanisms with promising effects in overcoming resistance to angiostatics. 

Angiogenesis can be revisited as a complex biological process with numerous compensatory pathways that can be activated, challenging the discovery of predictive biomarkers, since the cancer microenvironment and the complex milieu are difficult to classify and several actors are simultaneously shaping the key pro-angiogenic ecosystem. Bevacizumab represents the archetypic example of the various mechanisms of action, which may differ between cancer types and chemotherapy, unveiling the multifaceted functions in driving regression of existing tumor vasculature, halting new vessel growth, shaping the anti-permeability of surviving vasculature, and priming vessel normalization and co-option. Unfortunately, a poor correlation between response and survival exists, and the effects are mainly limited to PFS, and from a clinical trials standpoint, cross-over events at progression make identification of response criteria and biomarkers difficult [94]. Imaging has been employed in particular in glioblastoma by using MRI [95] in order to address the response to angiostatics. Another challenge for the investigation focused on predictive biomarkers is that bevacizumab is combined with standard chemotherapy, making it difficult to discriminate the response due to chemotherapeutic from the anti-angiogenic. However, multiple biomarkers from various locations within the tumor may play a role. Circulating angiogenic factors are deemed as the first in class indicators to predict outcome. Undeniably, high plasma VEGF is associated with worse outcomes regardless of bevacizumab therapy, being a prognostic marker rather than a predictive one [96]; nevertheless, while thinking about post-transcription regulation of VEGF, short isoforms (VEGF^110,120^) can easily diffuse in the tumor site. Contrariwise, longer isoforms (VEGF^165,189^) do not move far from the tumor. Thus, short VEGF isoforms could provide a better read-out of the tumor-derived VEGF, potentially impacting clinical prediction in metastatic breast, gastric, and pancreatic cancer. Even so, short VEGF isoforms hold prognostic but not predictive value in mCRC, mNSCLC, and mRCC [97], and it is necessary to bear in mind that in breast gastric and pancreatic cancer, EDTA was employed as sample buffer, although citrate has been utilized in the colorectal, lung, and renal malignancies. This technical characteristic might have impacted on the differences observed in the potentially predictive value [74,98]. The first biomarker-driven trial for anti-angiogenesis is de MERiDiAN GO25632 for metastatic breast cancer, in which patients receiving paclitaxel alone or in combination with bevacizumab were stratified according to short VEGF isoform levels [99,100]. Furthermore, a pro-angiogenic genetic signature has been used in other solid and haematological malignancies clinical and pre-clinical models, which characterized patients with good prognosis compared to others, showing that these patients can respond better to anti-angiogenic monotherapy rather than immunotherapy or other treatments even in combination [101,102,103]. Moreover, a compensatory upregulation of other angiogenic markers after VEGF inhibition emerged as a paradigmatic example of adaptive angiogenesis, because within a hypoxic environment besides VEGF, other factors such as PIGF, angiopoietins, and FGFs would be able to fuel into a vicious cycle [104]. The AFFIRM trial dynamically analyzed plasma markers while testing aflibercept in the first-line CRC, measuring 27 circulating angiogenic factors in plasma from mCRC patients receiving aflibercept. Baseline IL-8 and changes in IL-8 expression correlated with PFS in the aflibercept arm [105]. 

Multiple biomarkers from various locations within the tumor may play a role in the angiogenesis, because as early as 2012, Lambrechts et al. uncovered the genetic variant’s role in determining the VEGF circulation level [106], performing a very comprehensive SNPs analysis for all the anti-angiogenic factors, uncovering rs7993418 inVEGFR-1 to have a predictive value in bevacizumab response in pancreatic cancer patients enrolled in the AviTA study, with an improved survival. Pancreatic cancer patients with rs7993418 A allele have no improved OS in the placebo arm [106]. Moreover, VEGFR-1 rs7993418 C allele also correlates with reduced OS in 91 sunitinib-treated renal cell carcinoma, making it therefore tempting to speculate about a predictive role for the VEGF3-1 locus for tyrosine kinase inhibitors [107,108]. The basic functional output of the above-mentioned SNP in VEGFR-1 is that the patients who have high VEGFR-1 and high soluble VEGFR-1 (sVEGFR-1) have poor outcomes in different scenarios, such as bevacizumab [109,110,111,112,113] and cediranib [114,115]. Remarkably, immunohistochemistry confirmed these results on tumor tissue before treatment by showing high VEGFR-1 expression to be correlated with bevacizumab poor outcome in CRC (NO16966) and gastric cancer (AVAGAST) [116,117]. Other approaches investigated soluble neuropilin-1 (sNRP-1), which is a co-receptor for VEGF in mCRC. sNRP-1 levels predict response to bevacizumab and the VEGFR inhibitor Tivozanib in mCRC [118,119]. Figuratively, it can be conceivable that when we have high sVEGFR-1 or sNRP-1, VEGF is sequestered in a decoy receptor-dependent manner, and no response to anti-VEGF can be observed; contrariwise, in the case of low sVEGFR-1 or sNRP-1, a response to anti-VEGF would be likely. Thus, endogenous levels of soluble anti-angiogenic VEGF receptors are predictive of treatment outcome.

Tumor and stromal markers also play a major role in shaping the angiogenic landscape. In the frame of this thinking, low tumor NRP1 expression in mCRC (NO16966 trial) seems to be associated with improved PFS with similar findings for sNRP1 in various studies [120,121]. Tumor VEGF-A and NRP expression have also been evaluated in metastatic gastric cancer, confirming that low tumor NRP1 expression is associated with improved OS. A similar association was seen for PFS [117]. Newly emerging biomarkers in gastric carcinoma involving ramucirumab monotherapy pinpoint to immunohistochemistry for VEGFR-2 as a promising tool, since the patient showed a high VEGFR-2 score, even if no robust differences have been detected, likely due to a lack of statistical power [122]. Molecular subtyping holds the potential to overcome the basic pathological intra tumor phenotyping by providing a novel model aiming to develop the next generation of personalized approach. Hu et al. pioneered the identification of molecular dissection in angiogenesis phenotyping [123], and Sandmann et al. did so in angiostatic targeting, identifying four molecular subtypes in IDH1 wild-type glioblastoma based on gene expression data [124], looking at the response of patients as classified according to molecular subtypes, which have been developed from publicly available databases. Looking at PFS across the four identified subtypes, namely mesenchymal, proliferative, proneural, and unclassified, the mesenchymal and proneural subgroups exhibited a PFS benefit from bevacizumab. Mesenchymal GBM are highly angiogenic with stromal invasion, and proneural tend to be IDH wild-type tumors. Surprisingly, looking at the OS, the only molecular subtypes of GBM that had a OS benefit were shown in those subjects included in the proneural group, unveiling that not only the expression of angiogenic factors may be worth considering in terms of the predictive potential but also the downstream pathways affected by targeting the VEGF signaling [124]. These data prompted further investigation in the oncology field [125,126,127,128]. In colorectal cancer, several molecular subtypes have also been identified [129]. The mesenchymal subgroup, the one with worse prognosis, had a better response to regorafenib; nonetheless, these data need a statistically powered perspective validation [130].

To date, there is not a single biomarker that is consistent with all the trials tested, since response to anti-angiogenesis is a complex phenotype. A single biomarker might not be sufficient to capture the true heterogeneity of the response, and the response to anti-angiogenesis seems to be a continuum; thus, a panel of multiple markers may be necessary to cover the full spectrum, but application is clinically challenging. Biomarkers provide important information on how to overcome resistance to anti-angiogenic therapies by guiding the inhibition of multiple angiogenic factors, destabilizing resistant tumor vessels, and prompting a state-of-the art inhibition of immune-cell recruitment, assuming that the endothelial cells and vasculogenic function are gatekeepers of the immune patrolling and therefore envisioning a synergism with immunotherapeutics. 

## 7. What Can We Do and What Needs to Be Done?

**Figure 2:**
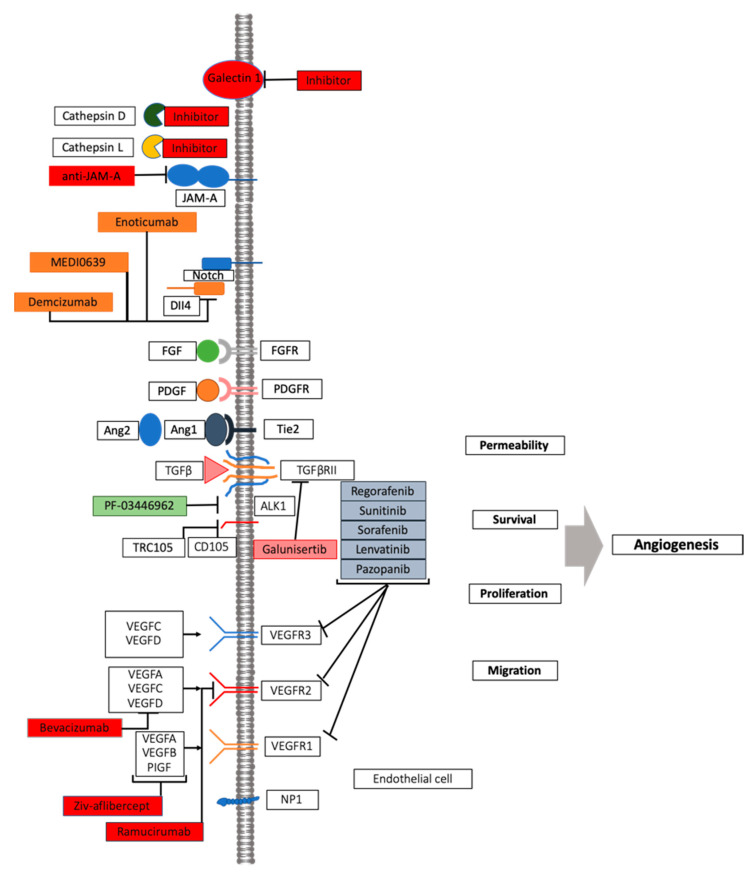
VEGF inhibition: mechanism of action and novel approaches. A number of pathway players represent druggable targets in angiogenesis process, including other members of VEGF, its receptor families, as well as other pro-angiogenic actors. See text for details.

The therapeutic effect of targeting a single angiogenic growth factor is limited due to intrinsic resistance as a consequence of redundancy in activated pathways or alternative growth factor signaling pathways [131]. The association of multiple anti-angiogenic molecules or a combination of anti-angiogenic drugs with other treatment regimens have been indicated as alternative therapeutic strategies to overcome resistance to anti-angiogenic therapies. Blocking the recruitment of monocytes-macrophages is another therapeutic strategy to overcome resistance to an anti-angiogenic therapy. In patients with solid tumors treated with carlumab, a human anti-CCL2 monoclonal antibody targeting the monocyte chemotactic protein 1 (MCP1), the growth of tumors has been delayed [132]. Treatment of pancreatic neuroendocrine tumors with Ang2 and VEGFR-2 blockers decreased Tie2 monocyte infiltration and suppressed revascularization and tumor progression [133], prompting next-generation anti-angiogenesis (Figure 2) [22,102,134]. The crosstalk between angiogenesis and immune cells explains the efficacy of combining anti-angiogenic drugs with immune check-point inhibitors. Increased PDL-1 expression has been observed in sunitinib-treated RCC cell lines and xenografts. Therefore, strategies targeting the immunosuppressive PD-1/PDL-1 signaling in anti-angiogenesis resistant tumors are emerging. The PD-1 inhibition via nivolumab monoclonal antibody has been approved in the treatment of advanced kidney cancer, and sunitinib–nivolumab and pazopanib–nivolumab combinations have been employed in subjects with metastatic cancer. Other strategies of combining immunotherapy and anti-angiogenics are currently being investigated [135]. Anti-angiogenic treatments are associated with increased local invasiveness and distant metastasis, as described for the first time in 2009 in the same issue of “Cancer Cell” by Ebos et al. [87] and Paez Ribes et al. [86] in different pre-clinical models. To overcome resistance by targeting increased tumor invasiveness and metastasis, different inhibitors of c-Met have been tested in preclinical studies [136,137]. Different factors are involved in acquired resistance, including decreased drug uptake, expression of new drug-efflux pumps, drug metabolism, tumor cell proliferation, and apoptotic mechanisms, therefore warranting further investigations in the field [16,72,138,139,140].

More precision and individualized approaches need to be tested in well-designed clinical trials—a challenge: a new model to the story that represents an archetype in the paradigm shift of anti-angiogenesis is represented by the immune system and its relationship with the vasculature [22]. Indeed, the immune cells distinguish between friend and foe by dynamic interactions often offering curative but also risk-associated therapy for many malignancies [141]. In preclinical and clinical models, anti-tumor mechanisms convey in a complex immune processes: the ultimate goal to translate findings into improved diagnostics and therapies has been realized while exploring the tumor angiogenesis induced by cancer-secreted factors/enzymes. A prototype of factors is Galectin-1, particularly over-expressed in tumor cells, and its expression correlates with malignancy and with acquisition of a metastatic phenotype [142]. When malignancies produce Galectin-1, it is in very high concentrations, and in its dimeric form and as a glycogen binding protein, a sugar binding protein and the binding to sugars on the surface of T-cells and immune cells and endothelial cells creates an immunosuppressive microenvironment on these cells. Thus, blocking galectin’s expression holds the promise to increase and unleash T-cell responses and shrink the tumor, also halting the number of blood vessels that are generated in the cancer microenvironment [143,144]. Cathepsins are lysosomal enzymes with enhanced concentration in malignant cells, in response to low oxygen levels and increased lactic acid concentration within the cancer niche [145,146]. Since the cathepsin and galectins axis may fuel the vicious cycle of a pro-vasculogenic environment [134], cathepsin/galectin targeting prompted novel strategies combining anti-angiogenic therapy and immunotherapy, with the potential to tip the balance of the tumor microenvironment and improve treatment response [141,147,148,149].

The major problem with the current use of anti-angiogenic therapies against tumors is the use of such therapies in the most inappropriate stage of disease. In this context, different therapeutic combinations may be personalized considering the current stage of tumor progression. Moreover, if we need to say why the effects of the anti-angiogenic drugs have been so disappointing so far, this is probably because of their use with a “carpet bombing” approach rather than using them according to the biology of the tumor. As recently suggested by Montemagno and Pagés, the better approach might be the following: “Instead of inhibiting several targets with several drugs, the ideal strategy relies on the use of one inhibitor targeting multiple hallmarks of cancers, i.e., tumor cell proliferation/stemness, angiogenesis, chronic inflammation, and immune tolerance.” [150].

## Figures and Tables

**Table 1:** Angiogenesis-targeting agents and their impact on survival.

Tumor Site	Name of the Study	Endpoint	Effect on OS Months (*p*-Value)	Effect on PFS Months (*p*-Value)
Prostate *	CALGB 90401	OS	2.4 (0.0001)	1.1 (0.18)
Pancreas *	AVITA	OS	1 (0.0002)	1.1 (0.21)
Lung *	ECOG E4599	OS	1.7 (<0.001)	2 (0.003)
Lung *	AVAIL	PFS, OS	0.6 (0.0003)	0.5 (0.2)
Kidney *	CALGB 90206	OS	3.3 (<0.001)	0.9 (0.097)
Breast *	ECOG E2100	PFS	5.9 (0.001)	1.5 (0.16)
Breast *	AVADO	PFS	1.9 (0.006)	−1.7 (0.85)
Breast *	RIBBON-1	PFS	2.9 (0.0002)	7.8 (0.27)
Gastric *	AVAGAST	OS	1.4 (0.0037)	2 (0.1)
Colorectal *	Hurwitz et al. (2008)	OS	4.4 (<0.001)	4.7 (<0.001)
Colorectal **	VELOUR	OS	2.23 (<0.001)	1.4 (0.0032)
Colorectal ^#^	RAISE	OS	1.2 (<0.0005)	1.6 (0.0219)
Colorectal ^##^	CORRECT	OS		1.4 (0.0052)

* Bevacizumab; ** Aflibercept; ^#^ Ramucirumab; ^##^ Regorafenib.

## Data Availability

Not applicable.

## References

[1] Marmé D. (2020). Tumor Angiogenesis A Key Target for Cancer Therapy.

[2] Hurwitz H., Fehrenbacher L., Novotny W., Cartwright T., Hainsworth J., Heim W., Berlin J., Baron A., Griffing S., Holmgren E. (2004). Bevacizumab plus Irinotecan, Fluorouracil, and Leucovorin for Metastatic Colorectal Cancer. N. Engl. J. Med..

[3] Hartmann J.T., Haap M., Kopp H.-G., Lipp H.-P. (2009). Tyrosine Kinase Inhibitors—A Review on Pharmacology, Metabolism and Side Effects. Curr. Drug Metab..

[4] Gaya A., Tse V. (2012). A Preclinical and Clinical Review of Aflibercept for the Management of Cancer. Cancer Treat. Rev..

[5] Van der Veldt A.A.M., Lubberink M., Bahce I., Walraven M., de Boer M.P., Greuter H.N.J.M., Hendrikse N.H., Eriksson J., Windhorst A.D., Postmus P.E. (2012). Rapid Decrease in Delivery of Chemotherapy to Tumors after Anti-VEGF Therapy: Implications for Scheduling of Anti-Angiogenic Drugs. Cancer Cell.

[6] Mancuso M.R., Davis R., Norberg S.M., O’Brien S., Sennino B., Nakahara T., Yao V.J., Inai T., Brooks P., Freimark B. (2006). Rapid Vascular Regrowth in Tumors after Reversal of VEGF Inhibition. J. Clin. Investig..

[7] Grothey A., Sugrue M.M., Purdie D.M., Dong W., Sargent D., Hedrick E., Kozloff M. (2008). Bevacizumab beyond First Progression Is Associated with Prolonged Overall Survival in Metastatic Colorectal Cancer: Results from a Large Observational Cohort Study (BRiTE). J. Clin. Oncol..

[8] Argentiero A., Solimando A.G., Brunetti O., Calabrese A., Pantano F., Iuliani M., Santini D., Silvestris N., Vacca A. (2019). Skeletal Metastases of Unknown Primary: Biological Landscape and Clinical Overview. Cancers.

[9] Antonio G., Oronzo B., Vito L., Angela C., Antonel-La A., Roberto C., Giovanni S.A., Antonella L. (2020). Immune System and Bone Microenvironment: Rationale for Targeted Cancer Therapies. Oncotarget.

[10] Kaplan R.N., Riba R.D., Zacharoulis S., Bramley A.H., Vincent L., Costa C., MacDonald D.D., Jin D.K., Shido K., Kerns S.A. (2005). VEGFR1-Positive Haematopoietic Bone Marrow Progenitors Initiate the Pre-Metastatic Niche. Nature.

[11] Argentiero A., Solimando A.G., Krebs M., Leone P., Susca N., Brunetti O., Racanelli V., Vacca A., Silvestris N. (2020). Anti-Angiogenesis and Immunotherapy: Novel Paradigms to Envision Tailored Approaches in Renal Cell-Carcinoma. J. Clin. Med..

[12] Shen C., Kaelin W.G. (2013). The VHL/HIF Axis in Clear Cell Renal Carcinoma. Semin. Cancer Biol..

[13] Goldman C.K., Kim J., Wong W.L., King V., Brock T., Gillespie G.Y. (1993). Epidermal Growth Factor Stimulates Vascular Endothelial Growth Factor Production by Human Malignant Glioma Cells: A Model of Glioblastoma Multiforme Pathophysiology. Mol. Biol. Cell.

[14] Van Beijnum J.R., Nowak-Sliwinska P., Huijbers E.J.M., Thijssen V.L., Griffioen A.W. (2015). The Great Escape; the Hallmarks of Resistance to Antiangiogenic Therapy. Pharm. Rev..

[15] Huijbers E.J.M., van Beijnum J.R., Thijssen V.L., Sabrkhany S., Nowak-Sliwinska P., Griffioen A.W. (2016). Role of the Tumor Stroma in Resistance to Anti-Angiogenic Therapy. Drug Resist. Updat..

[16] Gottesman M.M. (2002). Mechanisms of Cancer Drug Resistance. Annu. Rev. Med..

[17] Jain R.K. (2013). Normalizing Tumor Microenvironment to Treat Cancer: Bench to Bedside to Biomarkers. J. Clin. Oncol..

[18] Wilson W.R., Hay M.P. (2011). Targeting Hypoxia in Cancer Therapy. Nat. Rev. Cancer.

[19] Lu K.V., Chang J.P., Parachoniak C.A., Pandika M.M., Aghi M.K., Meyronet D., Isachenko N., Fouse S.D., Phillips J.J., Cheresh D.A. (2012). VEGF Inhibits Tumor Cell Invasion and Mesenchymal Transition through a MET/VEGFR2 Complex. Cancer Cell.

[20] Finger E.C., Giaccia A.J. (2010). Hypoxia, Inflammation, and the Tumor Microenvironment in Metastatic Disease. Cancer Metastasis Rev..

[21] Semenza G.L. (2014). Oxygen Sensing, Hypoxia-Inducible Factors, and Disease Pathophysiology. Annu. Rev. Pathol..

[22] Solimando A.G., Summa S.D., Vacca A., Ribatti D. (2020). Cancer-Associated Angiogenesis: The Endothelial Cell as a Checkpoint for Immunological Patrolling. Cancers.

[23] Sounni N.E., Cimino J., Blacher S., Primac I., Truong A., Mazzucchelli G., Paye A., Calligaris D., Debois D., De Tullio P. (2014). Blocking Lipid Synthesis Overcomes Tumor Regrowth and Metastasis after Antiangiogenic Therapy Withdrawal. Cell Metab..

[24] Vandyke K., Zeissig M.N., Hewett D.R., Martin S.K., Mrozik K.M., Cheong C.M., Diamond P., To L.B., Gronthos S., Peet D.J. (2017). HIF-2α Promotes Dissemination of Plasma Cells in Multiple Myeloma by Regulating CXCL12/CXCR4 and CCR1. Cancer Res..

[25] Solimando A.G., Vacca A., Ribatti D. (2020). A Comprehensive Biological and Clinical Perspective Can Drive a Patient-Tailored Approach to Multiple Myeloma: Bridging the Gaps between the Plasma Cell and the Neoplastic Niche. J. Oncol..

[26] Rankin E.B., Giaccia A.J. (2016). Hypoxic Control of Metastasis. Science.

[27] Shojaei F., Wu X., Zhong C., Yu L., Liang X.-H., Yao J., Blanchard D., Bais C., Peale F.V., van Bruggen N. (2007). Bv8 Regulates Myeloid-Cell-Dependent Tumour Angiogenesis. Nature.

[28] Ciesielski O., Biesiekierska M., Panthu B., Vialichka V., Pirola L., Balcerczyk A. (2020). The Epigenetic Profile of Tumor Endothelial Cells. Effects of Combined Therapy with Antiangiogenic and Epigenetic Drugs on Cancer Progression. Int. J. Mol. Sci..

[29] Luo M., Hou L., Li J., Shao S., Huang S., Meng D., Liu L., Feng L., Xia P., Qin T. (2016). VEGF/NRP-1axis Promotes Progression of Breast Cancer via Enhancement of Epithelial-Mesenchymal Transition and Activation of NF-ΚB and β-Catenin. Cancer Lett..

[30] Shimizu T., Ishida J., Kurozumi K., Ichikawa T., Otani Y., Oka T., Tomita Y., Hattori Y., Uneda A., Matsumoto Y. (2019). δ-Catenin Promotes Bevacizumab-Induced Glioma Invasion. Mol. Cancer Ther..

[31] Jahangiri A., De Lay M., Miller L.M., Carbonell W.S., Hu Y.-L., Lu K., Tom M.W., Paquette J., Tokuyasu T.A., Tsao S. (2013). Gene Expression Profile Identifies Tyrosine Kinase C-Met as a Targetable Mediator of Antiangiogenic Therapy Resistance. Clin. Cancer Res..

[32] Ribatti D., Djonov V. (2012). Intussusceptive Microvascular Growth in Tumors. Cancer Lett..

[33] Pezzella F., Ribatti D. (2020). Vascular Co-Option and Vasculogenic Mimicry Mediate Resistance to Antiangiogenic Strategies. Cancer Rep..

[34] Donnem T., Reynolds A.R., Kuczynski E.A., Gatter K., Vermeulen P.B., Kerbel R.S., Harris A.L., Pezzella F. (2018). Non-Angiogenic Tumours and Their Influence on Cancer Biology. Nat. Rev. Cancer.

[35] Kirschmann D.A., Seftor E.A., Hardy K.M., Seftor R.E.B., Hendrix M.J.C. (2012). Molecular Pathways: Vasculogenic Mimicry in Tumor Cells: Diagnostic and Therapeutic Implications. Clin. Cancer Res..

[36] Xu Y., Li Q., Li X.-Y., Yang Q.-Y., Xu W.-W., Liu G.-L. (2012). Short-Term Anti-Vascular Endothelial Growth Factor Treatment Elicits Vasculogenic Mimicry Formation of Tumors to Accelerate Metastasis. J. Exp. Clin. Cancer Res..

[37] Finley S.D., Chu L.-H., Popel A.S. (2015). Computational Systems Biology Approaches to Anti-Angiogenic Cancer Therapeutics. Drug Discov. Today.

[38] Costa M., Danesi R., Agen C., Di Paolo A., Basolo F., Del Bianchi S., Del Tacca M. (1994). MCF-10A Cells Infected with the Int-2 Oncogene Induce Angiogenesis in the Chick Chorioallantoic Membrane and in the Rat Mesentery. Cancer Res..

[39] Ferrara N., Carver-Moore K., Chen H., Dowd M., Lu L., O’Shea K.S., Powell-Braxton L., Hillan K.J., Moore M.W. (1996). Heterozygous Embryonic Lethality Induced by Targeted Inactivation of the VEGF Gene. Nature.

[40] Roy H., Bhardwaj S., Ylä-Herttuala S. (2006). Biology of Vascular Endothelial Growth Factors. FEBS Lett..

[41] Verdegem D., Moens S., Stapor P., Carmeliet P. (2014). Endothelial Cell Metabolism: Parallels and Divergences with Cancer Cell Metabolism. Cancer Metab..

[42] Aird W.C. (2012). Endothelial Cell Heterogeneity. Cold Spring Harb. Perspect. Med..

[43] Krebs M., Solimando A.G., Kalogirou C., Marquardt A., Frank T., Sokolakis I., Hatzichristodoulou G., Kneitz S., Bargou R., Kübler H. (2020). MiR-221-3p Regulates VEGFR2 Expression in High-Risk Prostate Cancer and Represents an Escape Mechanism from Sunitinib In Vitro. J. Clin. Med..

[44] Cai W., Chen X. (2008). Multimodality Molecular Imaging of Tumor Angiogenesis. J. Nucl. Med..

[45] Chouaib S., Messai Y., Couve S., Escudier B., Hasmim M., Noman M.Z. (2012). Hypoxia Promotes Tumor Growth in Linking Angiogenesis to Immune Escape. Front. Immunol..

[46] Yamashita-Kashima Y., Fujimoto-Ouchi K., Yorozu K., Kurasawa M., Yanagisawa M., Yasuno H., Mori K. (2012). Biomarkers for Antitumor Activity of Bevacizumab in Gastric Cancer Models. BMC Cancer.

[47] Sun X.-T., Yuan X.-W., Zhu H.-T., Deng Z.-M., Yu D.-C., Zhou X., Ding Y.-T. (2012). Endothelial Precursor Cells Promote Angiogenesis in Hepatocellular Carcinoma. World J. Gastroenterol..

[48] Backer M.V., Backer J.M. (2012). Imaging Key Biomarkers of Tumor Angiogenesis. Theranostics.

[49] Laking G.R., West C., Buckley D.L., Matthews J., Price P.M. (2006). Imaging Vascular Physiology to Monitor Cancer Treatment. Crit. Rev. Oncol. Hematol..

[50] Goel S., Duda D.G., Xu L., Munn L.L., Boucher Y., Fukumura D., Jain R.K. (2011). Normalization of the Vasculature for Treatment of Cancer and Other Diseases. Physiol. Rev..

[51] Linehan W.M., Vasselli J., Srinivasan R., Walther M.M., Merino M., Choyke P., Vocke C., Schmidt L., Isaacs J.S., Glenn G. (2004). Genetic Basis of Cancer of the Kidney: Disease-Specific Approaches to Therapy. Clin. Cancer Res..

[52] Wang Y., Li Z., Zhang H., Jin H., Sun L., Dong H., Xu M., Zhao P., Zhang B., Wang J. (2010). HIF-1α and HIF-2α Correlate with Migration and Invasion in Gastric Cancer. Cancer Biol. Ther..

[53] Tanigawa N., Amaya H., Matsumura M., Shimomatsuya T. (1997). Correlation between Expression of Vascular Endothelial Growth Factor and Tumor Vascularity, and Patient Outcome in Human Gastric Carcinoma. J. Clin. Oncol..

[54] Youssoufian H., Hicklin D.J., Rowinsky E.K. (2007). Review: Monoclonal Antibodies to the Vascular Endothelial Growth Factor Receptor-2 in Cancer Therapy. Clin. Cancer Res..

[55] Maeda K., Chung Y.S., Ogawa Y., Takatsuka S., Kang S.M., Ogawa M., Sawada T., Sowa M. (1996). Prognostic Value of Vascular Endothelial Growth Factor Expression in Gastric Carcinoma. Cancer.

[56] Suzuki S., Dobashi Y., Hatakeyama Y., Tajiri R., Fujimura T., Heldin C.H., Ooi A. (2010). Clinicopathological Significance of Platelet-Derived Growth Factor (PDGF)-B and Vascular Endothelial Growth Factor-A Expression, PDGF Receptor-β Phosphorylation, and Microvessel Density in Gastric Cancer. BMC Cancer.

[57] Villarejo-Campos P., Padilla-Valverde D., Martin R.M., Menéndez-Sánchez P., Cubo-Cintas T., Bondia-Navarro J.A., Fernández J.M. (2013). Serum VEGF and VEGF-C Values before Surgery and after Postoperative Treatment in Gastric Cancer. Clin. Transl. Oncol..

[58] Wang X., Chen X., Fang J., Yang C. (2013). Overexpression of Both VEGF-A and VEGF-C in Gastric Cancer Correlates with Prognosis, and Silencing of Both Is Effective to Inhibit Cancer Growth. Int. J. Clin. Exp. Pathol..

[59] Yanagisawa M., Yorozu K., Kurasawa M., Nakano K., Furugaki K., Yamashita Y., Mori K., Fujimoto-Ouchi K. (2010). Bevacizumab Improves the Delivery and Efficacy of Paclitaxel. Anticancer Drugs.

[60] Kamiyama H., Takano S., Tsuboi K., Matsumura A. (2005). Anti-Angiogenic Effects of SN38 (Active Metabolite of Irinotecan): Inhibition of Hypoxia-Inducible Factor 1 Alpha (HIF-1alpha)/Vascular Endothelial Growth Factor (VEGF) Expression of Glioma and Growth of Endothelial Cells. J. Cancer Res. Clin. Oncol..

[61] Giuliano S., Pagès G. (2013). Mechanisms of Resistance to Anti-Angiogenesis Therapies. Biochimie.

[62] Russano M., Napolitano A., Ribelli G., Iuliani M., Simonetti S., Citarella F., Pantano F., Dell’Aquila E., Anesi C., Silvestris N. (2020). Liquid Biopsy and Tumor Heterogeneity in Metastatic Solid Tumors: The Potentiality of Blood Samples. J. Exp. Clin. Cancer Res..

[63] Croci D.O., Cerliani J.P., Dalotto-Moreno T., Méndez-Huergo S.P., Mascanfroni I.D., Dergan-Dylon S., Toscano M.A., Caramelo J.J., García-Vallejo J.J., Ouyang J. (2014). Glycosylation-Dependent Lectin-Receptor Interactions Preserve Angiogenesis in Anti-VEGF Refractory Tumors. Cell.

[64] Bergers G., Hanahan D. (2008). Modes of Resistance to Anti-Angiogenic Therapy. Nat. Rev. Cancer.

[65] Hashizume H., Falcón B.L., Kuroda T., Baluk P., Coxon A., Yu D., Bready J.V., Oliner J.D., McDonald D.M. (2010). Complementary Actions of Inhibitors of Angiopoietin-2 and VEGF on Tumor Angiogenesis and Growth. Cancer Res..

[66] Lamanuzzi A., Saltarella I., Desantis V., Frassanito M.A., Leone P., Racanelli V., Nico B., Ribatti D., Ditonno P., Prete M. (2018). Inhibition of MTOR Complex 2 Restrains Tumor Angiogenesis in Multiple Myeloma. Oncotarget.

[67] Rao L., Giannico D., Leone P., Solimando A.G., Maiorano E., Caporusso C., Duda L., Tamma R., Mallamaci R., Susca N. (2020). HB-EGF-EGFR Signaling in Bone Marrow Endothelial Cells Mediates Angiogenesis Associated with Multiple Myeloma. Cancers.

[68] Rao L., De Veirman K., Giannico D., Saltarella I., Desantis V., Frassanito M.A., Solimando A.G., Ribatti D., Prete M., Harstrick A. (2018). Targeting Angiogenesis in Multiple Myeloma by the VEGF and HGF Blocking DARPin^®^ Protein MP0250: A Preclinical Study. Oncotarget.

[69] Leone P., Buonavoglia A., Fasano R., Solimando A.G., De Re V., Cicco S., Vacca A., Racanelli V. (2019). Insights into the Regulation of Tumor Angiogenesis by Micro-RNAs. J. Clin. Med..

[70] Leone P., Solimando A.G., Malerba E., Fasano R., Buonavoglia A., Pappagallo F., De Re V., Argentiero A., Silvestris N., Vacca A. (2020). Actors on the Scene: Immune Cells in the Myeloma Niche. Front. Oncol..

[71] Hughes P.E., Caenepeel S., Wu L.C. (2016). Targeted Therapy and Checkpoint Immunotherapy Combinations for the Treatment of Cancer. Trends Immunol..

[72] Jridi I., Catacchio I., Majdoub H., Shahbazzadeh D., El Ayeb M., Frassanito M.A., Solimando A.G., Ribatti D., Vacca A., Borchani L. (2017). The Small Subunit of Hemilipin2, a New Heterodimeric Phospholipase A2 from Hemiscorpius Lepturus Scorpion Venom, Mediates the Antiangiogenic Effect of the Whole Protein. Toxicon.

[73] Shanmugam M.K., Warrier S., Kumar A.P., Sethi G., Arfuso F. (2017). Potential Role of Natural Compounds as Anti-Angiogenic Agents in Cancer. Curr. Vasc. Pharmacol..

[74] Jayson G.C., Kerbel R., Ellis L.M., Harris A.L. (2016). Antiangiogenic Therapy in Oncology: Current Status and Future Directions. Lancet.

[75] Tartour E., Pere H., Maillere B., Terme M., Merillon N., Taieb J., Sandoval F., Quintin-Colonna F., Lacerda K., Karadimou A. (2011). Angiogenesis and Immunity: A Bidirectional Link Potentially Relevant for the Monitoring of Antiangiogenic Therapy and the Development of Novel Therapeutic Combination with Immunotherapy. Cancer Metastasis Rev..

[76] Korc M. (2003). Pathways for Aberrant Angiogenesis in Pancreatic Cancer. Mol. Cancer.

[77] Porcelli L., Iacobazzi R.M., Di Fonte R., Serratì S., Intini A., Solimando A.G., Brunetti O., Calabrese A., Leonetti F., Azzariti A. (2019). CAFs and TGF-β Signaling Activation by Mast Cells Contribute to Resistance to Gemcitabine/Nabpaclitaxel in Pancreatic Cancer. Cancers.

[78] Argentiero A., De Summa S., Di Fonte R., Iacobazzi R.M., Porcelli L., Da Vià M., Brunetti O., Azzariti A., Silvestris N., Solimando A.G. (2019). Gene Expression Comparison between the Lymph Node-Positive and -Negative Reveals a Peculiar Immune Microenvironment Signature and a Theranostic Role for WNT Targeting in Pancreatic Ductal Adenocarcinoma: A Pilot Study. Cancers.

[79] Javadrashid D., Baghbanzadeh A., Derakhshani A., Leone P., Silvestris N., Racanelli V., Solimando A.G., Baradaran B. (2021). Pancreatic Cancer Signaling Pathways, Genetic Alterations, and Tumor Microenvironment: The Barriers Affecting the Method of Treatment. Biomedicines.

[80] Jiang H., Hegde S., Knolhoff B.L., Zhu Y., Herndon J.M., Meyer M.A., Nywening T.M., Hawkins W.G., Shapiro I.M., Weaver D.T. (2016). Targeting Focal Adhesion Kinase Renders Pancreatic Cancers Responsive to Checkpoint Immunotherapy. Nat. Med..

[81] Haibe Y., Kreidieh M., El Hajj H., Khalifeh I., Mukherji D., Temraz S., Shamseddine A. (2020). Resistance Mechanisms to Anti-Angiogenic Therapies in Cancer. Front. Oncol..

[82] Bertolini F., Paul S., Mancuso P., Monestiroli S., Gobbi A., Shaked Y., Kerbel R.S. (2003). Maximum Tolerable Dose and Low-Dose Metronomic Chemotherapy Have Opposite Effects on the Mobilization and Viability of Circulating Endothelial Progenitor Cells. Cancer Res..

[83] Hellström M., Kalén M., Lindahl P., Abramsson A., Betsholtz C. (1999). Role of PDGF-B and PDGFR-Beta in Recruitment of Vascular Smooth Muscle Cells and Pericytes during Embryonic Blood Vessel Formation in the Mouse. Development.

[84] Gerhardt H., Betsholtz C. (2003). Endothelial-Pericyte Interactions in Angiogenesis. Cell Tissue Res..

[85] Chintalgattu V., Rees M.L., Culver J.C., Goel A., Jiffar T., Zhang J., Dunner K., Pati S., Bankson J.A., Pasqualini R. (2013). Coronary Microvascular Pericytes Are the Cellular Target of Sunitinib Malate-Induced Cardiotoxicity. Sci. Transl. Med..

[86] Pàez-Ribes M., Allen E., Hudock J., Takeda T., Okuyama H., Viñals F., Inoue M., Bergers G., Hanahan D., Casanovas O. (2009). Antiangiogenic Therapy Elicits Malignant Progression of Tumors to Increased Local Invasion and Distant Metastasis. Cancer Cell.

[87] Ebos J.M.L., Lee C.R., Cruz-Munoz W., Bjarnason G.A., Christensen J.G., Kerbel R.S. (2009). Accelerated Metastasis after Short-Term Treatment with a Potent Inhibitor of Tumor Angiogenesis. Cancer Cell.

[88] Loges S., Mazzone M., Hohensinner P., Carmeliet P. (2009). Silencing or Fueling Metastasis with VEGF Inhibitors: Antiangiogenesis Revisited. Cancer Cell.

[89] Zuniga R.M., Torcuator R., Jain R., Anderson J., Doyle T., Ellika S., Schultz L., Mikkelsen T. (2009). Efficacy, Safety and Patterns of Response and Recurrence in Patients with Recurrent High-Grade Gliomas Treated with Bevacizumab plus Irinotecan. J. Neurooncol..

[90] Todorova D., Simoncini S., Lacroix R., Sabatier F., Dignat-George F. (2017). Extracellular Vesicles in Angiogenesis. Circ. Res..

[91] Giuliano S., Cormerais Y., Dufies M., Grépin R., Colosetti P., Belaid A., Parola J., Martin A., Lacas-Gervais S., Mazure N.M. (2015). Resistance to Sunitinib in Renal Clear Cell Carcinoma Results from Sequestration in Lysosomes and Inhibition of the Autophagic Flux. Autophagy.

[92] Wiedmer T., Blank A., Pantasis S., Normand L., Bill R., Krebs P., Tschan M.P., Marinoni I., Perren A. (2017). Autophagy Inhibition Improves Sunitinib Efficacy in Pancreatic Neuroendocrine Tumors via a Lysosome-Dependent Mechanism. Mol. Cancer Ther..

[93] Jain R.K., Duda D.G., Willett C.G., Sahani D.V., Zhu A.X., Loeffler J.S., Batchelor T.T., Sorensen A.G. (2009). Biomarkers of Response and Resistance to Antiangiogenic Therapy. Nat. Rev. Clin. Oncol..

[94] Lambrechts D., Lenz H.-J., de Haas S., Carmeliet P., Scherer S.J. (2013). Markers of Response for the Antiangiogenic Agent Bevacizumab. J. Clin. Oncol..

[95] Arevalo O.D., Soto C., Rabiei P., Kamali A., Ballester L.Y., Esquenazi Y., Zhu J.-J., Riascos R.F. (2019). Assessment of Glioblastoma Response in the Era of Bevacizumab: Longstanding and Emergent Challenges in the Imaging Evaluation of Pseudoresponse. Front. Neurol..

[96] Hegde P.S., Jubb A.M., Chen D., Li N.F., Meng Y.G., Bernaards C., Elliott R., Scherer S.J., Chen D.S. (2013). Predictive Impact of Circulating Vascular Endothelial Growth Factor in Four Phase III Trials Evaluating Bevacizumab. Clin. Cancer Res..

[97] Miles D.W., de Haas S.L., Dirix L.Y., Romieu G., Chan A., Pivot X., Tomczak P., Provencher L., Cortés J., Delmar P.R. (2013). Biomarker Results from the AVADO Phase 3 Trial of First-Line Bevacizumab plus Docetaxel for HER2-Negative Metastatic Breast Cancer. Br. J. Cancer.

[98] Gianni L., Romieu G.H., Lichinitser M., Serrano S.V., Mansutti M., Pivot X., Mariani P., Andre F., Chan A., Lipatov O. (2013). AVEREL: A Randomized Phase III Trial Evaluating Bevacizumab in Combination with Docetaxel and Trastuzumab as First-Line Therapy for HER2-Positive Locally Recurrent/Metastatic Breast Cancer. J. Clin. Oncol..

[99] Miles D., Cameron D., Bondarenko I., Manzyuk L., Alcedo J.C., Lopez R.I., Im S.-A., Canon J.-L., Shparyk Y., Yardley D.A. (2017). Bevacizumab plus Paclitaxel versus Placebo plus Paclitaxel as First-Line Therapy for HER2-Negative Metastatic Breast Cancer (MERiDiAN): A Double-Blind Placebo-Controlled Randomised Phase III Trial with Prospective Biomarker Evaluation. Eur. J. Cancer.

[100] Masuda N., Takahashi M., Nakagami K., Okumura Y., Nakayama T., Sato N., Kanatani K., Tajima K., Kashiwaba M. (2017). First-Line Bevacizumab plus Paclitaxel in Japanese Patients with HER2-Negative Metastatic Breast Cancer: Subgroup Results from the Randomized Phase III MERiDiAN Trial. Jpn. J. Clin. Oncol..

[101] McDermott D.F., Huseni M.A., Atkins M.B., Motzer R.J., Rini B.I., Escudier B., Fong L., Joseph R.W., Pal S.K., Reeves J.A. (2018). Clinical Activity and Molecular Correlates of Response to Atezolizumab Alone or in Combination with Bevacizumab versus Sunitinib in Renal Cell Carcinoma. Nat. Med..

[102] Solimando A.G., Da Vià M.C., Leone P., Borrelli P., Croci G.A., Tabares P., Brandl A., Di Lernia G., Bianchi F.P., Tafuri S. (2021). Halting the Vicious Cycle within the Multiple Myeloma Ecosystem: Blocking JAM-A on Bone Marrow Endothelial Cells Restores the Angiogenic Homeostasis and Suppresses Tumor Progression. Haematologica.

[103] Solimando A.G., Annese T., Tamma R., Ingravallo G., Maiorano E., Vacca A., Specchia G., Ribatti D. (2020). New Insights into Diffuse Large B-Cell Lymphoma Pathobiology. Cancers.

[104] Rapisarda A., Melillo G. (2009). Role of the Hypoxic Tumor Microenvironment in the Resistance to Anti-Angiogenic Therapies. Drug Resist. Updat..

[105] Lambrechts D., Thienpont B., Thuillier V., Sagaert X., Moisse M., Peuteman G., Pericay C., Folprecht G., Zalcberg J., Zilocchi C. (2015). Evaluation of Efficacy and Safety Markers in a Phase II Study of Metastatic Colorectal Cancer Treated with Aflibercept in the First-Line Setting. Br. J. Cancer.

[106] Lambrechts D., Claes B., Delmar P., Reumers J., Mazzone M., Yesilyurt B.T., Devlieger R., Verslype C., Tejpar S., Wildiers H. (2012). VEGF Pathway Genetic Variants as Biomarkers of Treatment Outcome with Bevacizumab: An Analysis of Data from the AViTA and AVOREN Randomised Trials. Lancet Oncol..

[107] Beuselinck B., Karadimou A., Lambrechts D., Claes B., Wolter P., Couchy G., Berkers J., Paridaens R., Schöffski P., Méjean A. (2013). Single-Nucleotide Polymorphisms Associated with Outcome in Metastatic Renal Cell Carcinoma Treated with Sunitinib. Br. J. Cancer.

[108] Beuselinck B., Karadimou A., Lambrechts D., Claes B., Wolter P., Couchy G., Berkers J., van Poppel H., Paridaens R., Schöffski P. (2014). VEGFR1 Single Nucleotide Polymorphisms Associated with Outcome in Patients with Metastatic Renal Cell Carcinoma Treated with Sunitinib—A Multicentric Retrospective Analysis. Acta Oncol..

[109] Duda D.G., Willett C.G., Ancukiewicz M., di Tomaso E., Shah M., Czito B.G., Bentley R., Poleski M., Lauwers G.Y., Carroll M. (2010). Plasma Soluble VEGFR-1 Is a Potential Dual Biomarker of Response and Toxicity for Bevacizumab with Chemoradiation in Locally Advanced Rectal Cancer. Oncologist.

[110] Willett C.G., Duda D.G., di Tomaso E., Boucher Y., Ancukiewicz M., Sahani D.V., Lahdenranta J., Chung D.C., Fischman A.J., Lauwers G.Y. (2009). Efficacy, Safety, and Biomarkers of Neoadjuvant Bevacizumab, Radiation Therapy, and Fluorouracil in Rectal Cancer: A Multidisciplinary Phase II Study. J. Clin. Oncol..

[111] Heist R.S., Duda D.G., Sahani D.V., Ancukiewicz M., Fidias P., Sequist L.V., Temel J.S., Shaw A.T., Pennell N.A., Neal J.W. (2015). Improved Tumor Vascularization after Anti-VEGF Therapy with Carboplatin and Nab-Paclitaxel Associates with Survival in Lung Cancer. Proc. Natl. Acad. Sci. USA.

[112] Aoyagi Y., Iinuma H., Horiuchi A., Shimada R., Watanabe T. (2010). Association of Plasma VEGF-A, Soluble VEGFR-1 and VEGFR-2 Levels and Clinical Response and Survival in Advanced Colorectal Cancer Patients Receiving Bevacizumab with Modified FOLFOX6. Oncol. Lett..

[113] Tolaney S.M., Boucher Y., Duda D.G., Martin J.D., Seano G., Ancukiewicz M., Barry W.T., Goel S., Lahdenrata J., Isakoff S.J. (2015). Role of Vascular Density and Normalization in Response to Neoadjuvant Bevacizumab and Chemotherapy in Breast Cancer Patients. Proc. Natl. Acad. Sci. USA.

[114] Zhu A.X., Ancukiewicz M., Supko J.G., Sahani D.V., Blaszkowsky L.S., Meyerhardt J.A., Abrams T.A., McCleary N.J., Bhargava P., Muzikansky A. (2013). Efficacy, Safety, Pharmacokinetics, and Biomarkers of Cediranib Monotherapy in Advanced Hepatocellular Carcinoma: A Phase II Study. Clin. Cancer Res..

[115] Batchelor T.T., Gerstner E.R., Emblem K.E., Duda D.G., Kalpathy-Cramer J., Snuderl M., Ancukiewicz M., Polaskova P., Pinho M.C., Jennings D. (2013). Improved Tumor Oxygenation and Survival in Glioblastoma Patients Who Show Increased Blood Perfusion after Cediranib and Chemoradiation. Proc. Natl. Acad. Sci. USA.

[116] Weickhardt A.J., Williams D.S., Lee C.K., Chionh F., Simes J., Murone C., Wilson K., Parry M.M., Asadi K., Scott A.M. (2015). Vascular Endothelial Growth Factor D Expression Is a Potential Biomarker of Bevacizumab Benefit in Colorectal Cancer. Br. J. Cancer.

[117] Van Cutsem E., de Haas S., Kang Y.-K., Ohtsu A., Tebbutt N.C., Ming Xu J., Peng Yong W., Langer B., Delmar P., Scherer S.J. (2012). Bevacizumab in Combination with Chemotherapy as First-Line Therapy in Advanced Gastric Cancer: A Biomarker Evaluation from the AVAGAST Randomized Phase III Trial. J. Clin. Oncol..

[118] Benson A.B., Kiss I., Bridgewater J., Eskens F.A.L.M., Sasse C., Vossen S., Chen J., Van Sant C., Ball H.A., Keating A. (2016). BATON-CRC: A Phase II Randomized Trial Comparing Tivozanib Plus MFOLFOX6 with Bevacizumab Plus MFOLFOX6 in Stage IV Metastatic Colorectal Cancer. Clin. Cancer Res..

[119] Ou J.-J., Wei X., Peng Y., Zha L., Zhou R.-B., Shi H., Zhou Q., Liang H.-J. (2015). Neuropilin-2 Mediates Lymphangiogenesis of Colorectal Carcinoma via a VEGFC/VEGFR3 Independent Signaling. Cancer Lett..

[120] Miao H.Q., Lee P., Lin H., Soker S., Klagsbrun M. (2000). Neuropilin-1 Expression by Tumor Cells Promotes Tumor Angiogenesis and Progression. FASEB J..

[121] Oh H., Takagi H., Otani A., Koyama S., Kemmochi S., Uemura A., Honda Y. (2002). Selective Induction of Neuropilin-1 by Vascular Endothelial Growth Factor (VEGF): A Mechanism Contributing to VEGF-Induced Angiogenesis. Proc. Natl. Acad. Sci. USA.

[122] Fuchs C.S., Tabernero J., Tomášek J., Chau I., Melichar B., Safran H., Tehfe M.A., Filip D., Topuzov E., Schlittler L. (2016). Biomarker Analyses in REGARD Gastric/GEJ Carcinoma Patients Treated with VEGFR2-Targeted Antibody Ramucirumab. Br. J. Cancer.

[123] Hu J., Bianchi F., Ferguson M., Cesario A., Margaritora S., Granone P., Goldstraw P., Tetlow M., Ratcliffe C., Nicholson A.G. (2005). Gene Expression Signature for Angiogenic and Nonangiogenic Non-Small-Cell Lung Cancer. Oncogene.

[124] Sandmann T., Bourgon R., Garcia J., Li C., Cloughesy T., Chinot O.L., Wick W., Nishikawa R., Mason W., Henriksson R. (2015). Patients With Proneural Glioblastoma May Derive Overall Survival Benefit From the Addition of Bevacizumab to First-Line Radiotherapy and Temozolomide: Retrospective Analysis of the AVAglio Trial. J. Clin. Oncol..

[125] Becht E., de Reyniès A., Giraldo N.A., Pilati C., Buttard B., Lacroix L., Selves J., Sautès-Fridman C., Laurent-Puig P., Fridman W.H. (2016). Immune and Stromal Classification of Colorectal Cancer Is Associated with Molecular Subtypes and Relevant for Precision Immunotherapy. Clin. Cancer Res..

[126] Bentink S., Haibe-Kains B., Risch T., Fan J.-B., Hirsch M.S., Holton K., Rubio R., April C., Chen J., Wickham-Garcia E. (2012). Angiogenic MRNA and MicroRNA Gene Expression Signature Predicts a Novel Subtype of Serous Ovarian Cancer. PLoS ONE.

[127] Escudier B., Motzer R.J., Tannir N.M., Porta C., Tomita Y., Maurer M.A., McHenry M.B., Rini B.I. (2020). Efficacy of Nivolumab plus Ipilimumab According to Number of IMDC Risk Factors in CheckMate 214. Eur. Urol..

[128] Marquardt A., Solimando A.G., Kerscher A., Bittrich M., Kalogirou C., Kübler H., Rosenwald A., Bargou R., Kollmannsberger P., Schilling B. (2021). Subgroup-Independent Mapping of Renal Cell Carcinoma-Machine Learning Reveals Prognostic Mitochondrial Gene Signature Beyond Histopathologic Boundaries. Front. Oncol..

[129] Marisa L., de Reyniès A., Duval A., Selves J., Gaub M.P., Vescovo L., Etienne-Grimaldi M.-C., Schiappa R., Guenot D., Ayadi M. (2013). Gene Expression Classification of Colon Cancer into Molecular Subtypes: Characterization, Validation, and Prognostic Value. PLoS Med..

[130] Guinney J., Dienstmann R., Wang X., de Reyniès A., Schlicker A., Soneson C., Marisa L., Roepman P., Nyamundanda G., Angelino P. (2015). The Consensus Molecular Subtypes of Colorectal Cancer. Nat. Med..

[131] Ribatti D. (2011). Novel Angiogenesis Inhibitors: Addressing the Issue of Redundancy in the Angiogenic Signaling Pathway. Cancer Treat. Rev..

[132] Sandhu S.K., Papadopoulos K., Fong P.C., Patnaik A., Messiou C., Olmos D., Wang G., Tromp B.J., Puchalski T.A., Balkwill F. (2013). A First-in-Human, First-in-Class, Phase I Study of Carlumab (CNTO 888), a Human Monoclonal Antibody against CC-Chemokine Ligand 2 in Patients with Solid Tumors. Cancer Chemother. Pharmacol..

[133] Rigamonti N., Kadioglu E., Keklikoglou I., Wyser Rmili C., Leow C.C., De Palma M. (2014). Role of Angiopoietin-2 in Adaptive Tumor Resistance to VEGF Signaling Blockade. Cell Rep..

[134] Pranjol M.Z.I., Zinovkin D.A., Maskell A.R.T., Stephens L.J., Achinovich S.L., Los’ D.M., Nadyrov E.A., Hannemann M., Gutowski N.J., Whatmore J.L. (2019). Cathepsin L-Induced Galectin-1 May Act as a Proangiogenic Factor in the Metastasis of High-Grade Serous Carcinoma. J. Transl. Med..

[135] Ciciola P., Cascetta P., Bianco C., Formisano L., Bianco R. (2020). Combining Immune Checkpoint Inhibitors with Anti-Angiogenic Agents. J. Clin. Med..

[136] Sennino B., Ishiguro-Oonuma T., Wei Y., Naylor R.M., Williamson C.W., Bhagwandin V., Tabruyn S.P., You W.-K., Chapman H.A., Christensen J.G. (2012). Suppression of Tumor Invasion and Metastasis by Concurrent Inhibition of C-Met and VEGF Signaling in Pancreatic Neuroendocrine Tumors. Cancer Discov..

[137] Shojaei F., Simmons B.H., Lee J.H., Lappin P.B., Christensen J.G. (2012). HGF/c-Met Pathway Is One of the Mediators of Sunitinib-Induced Tumor Cell Type-Dependent Metastasis. Cancer Lett..

[138] Desantis V., Frassanito M.A., Tamma R., Saltarella I., Di Marzo L., Lamanuzzi A., Solimando A.G., Ruggieri S., Annese T., Nico B. (2018). Rhu-Epo down-Regulates pro-Tumorigenic Activity of Cancer-Associated Fibroblasts in Multiple Myeloma. Ann. Hematol..

[139] Lamanuzzi A., Saltarella I., Frassanito M.A., Ribatti D., Melaccio A., Desantis V., Solimando A.G., Ria R., Vacca A. (2021). Thrombopoietin Promotes Angiogenesis and Disease Progression in Patients with Multiple Myeloma. Am. J. Pathol..

[140] Leone P., Di Lernia G., Solimando A.G., Cicco S., Saltarella I., Lamanuzzi A., Ria R., Frassanito M.A., Ponzoni M., Ditonno P. (2019). Bone Marrow Endothelial Cells Sustain a Tumor-Specific CD8+ T Cell Subset with Suppressive Function in Myeloma Patients. Oncoimmunology.

[141] Alard E., Butnariu A.-B., Grillo M., Kirkham C., Zinovkin D.A., Newnham L., Macciochi J., Pranjol M.Z.I. (2020). Advances in Anti-Cancer Immunotherapy: Car-T Cell, Checkpoint Inhibitors, Dendritic Cell Vaccines, and Oncolytic Viruses, and Emerging Cellular and Molecular Targets. Cancers.

[142] Dalotto-Moreno T., Croci D.O., Cerliani J.P., Martinez-Allo V.C., Dergan-Dylon S., Méndez-Huergo S.P., Stupirski J.C., Mazal D., Osinaga E., Toscano M.A. (2013). Targeting Galectin-1 Overcomes Breast Cancer-Associated Immunosuppression and Prevents Metastatic Disease. Cancer Res..

[143] Ruvolo P.P. (2016). Galectin 3 as a Guardian of the Tumor Microenvironment. Biochim. Biophys. Acta.

[144] Girotti M.R., Salatino M., Dalotto-Moreno T., Rabinovich G.A. (2020). Sweetening the Hallmarks of Cancer: Galectins as Multifunctional Mediators of Tumor Progression. J. Exp. Med..

[145] Levicar N., Strojnik T., Kos J., Dewey R.A., Pilkington G.J., Lah T.T. (2002). Lysosomal Enzymes, Cathepsins in Brain Tumour Invasion. J. Neurooncol..

[146] Nagaraj N.S., Vigneswaran N., Zacharias W. (2007). Hypoxia Inhibits TRAIL-Induced Tumor Cell Apoptosis: Involvement of Lysosomal Cathepsins. Apoptosis.

[147] Orozco C.A., Martinez-Bosch N., Guerrero P.E., Vinaixa J., Dalotto-Moreno T., Iglesias M., Moreno M., Djurec M., Poirier F., Gabius H.-J. (2018). Targeting Galectin-1 Inhibits Pancreatic Cancer Progression by Modulating Tumor-Stroma Crosstalk. Proc. Natl. Acad. Sci. USA.

[148] Baldwin E.T., Bhat T.N., Gulnik S., Hosur M.V., Sowder R.C., Cachau R.E., Collins J., Silva A.M., Erickson J.W. (1993). Crystal Structures of Native and Inhibited Forms of Human Cathepsin D: Implications for Lysosomal Targeting and Drug Design. Proc. Natl. Acad. Sci. USA.

[149] Sudhan D.R., Siemann D.W. (2015). Cathepsin L Targeting in Cancer Treatment. Pharmacol. Ther..

[150] Montemagno C., Pagès G. (2020). Resistance to Anti-Angiogenic Therapies: A Mechanism Depending on the Time of Exposure to the Drugs. Front. Cell Dev. Biol..

